# The association between motor coordination, physical fitness, and cognitive function in preschool children: physical fitness as a key bridge between motor coordination and executive function

**DOI:** 10.3389/fpubh.2026.1795664

**Published:** 2026-04-08

**Authors:** Hejie Zhang, Deqiang Zhao, Xiaoxiao Chen, Aoyu Zhang, Chunmiao Wang, Jiaxin Chen, Haixia Hu, Yanfeng Zhang

**Affiliations:** 1 China Institute of Sport Science, Beijing, China; 2 School of Physical Education and Sports Rehabilitation, Jinzhou Medical University, Jinzhou, Liaoning, China; 3 Tianshi College of Tianjin, School of Medicine, Tianjin, China; 4 Aiyoudong Children and Youth Sports Health Research Institute, Wei fang, Shandong, China

**Keywords:** cognitive function, mediation effect, motor coordination, physical fitness, preschool children

## Abstract

The preschool period (3–6 years) is a critical window for the development of motor abilities, physical fitness, and executive function. Objective: This study aimed to investigate the association between motor coordination (MC) and physical fitness (PFI), examine their independent predictive effects on three core executive functions (cognitive flexibility, inhibitory control, working memory), and verify the mediating role of PFI.

**Methods:**

A cross-sectional study was conducted with children aged 3–6 years. Motor coordination (MC) was assessed using the MABC-2, physical fitness (PFI) via the Chinese National Physical Fitness Measurement Standards Manual for Preschool Children, and cognitive functions through validated behavioral tasks. Pearson correlation, hierarchical regression, and Bootstrap mediation analyses were employed.

**Results:**

A total of 713 children (386 boys, 327 girls) were included in the analysis. MC was significantly correlated with PFI (*r* = 0.443, *p* < 0.01). Both MC and PFI were positively correlated with all cognitive functions (rs: 0.145–0.250). Regression analysis showed MC and PFI independently predicted cognitive flexibility (*β*_MC = 0.141, *p* = 0.001; *β*_PFI = 0.106, *p* = 0.015) and inhibitory control (*β*_MC = 0.120, *p* = 0.003; *β*_PFI = 0.098, *p* = 0.025). Only PFI predicted working memory (*β* = 0.093, *p* = 0.035). PFI significantly mediated the relationship between MC and all cognitive functions, acting as a complete mediator for working memory (indirect effect: 46.9%), and a partial mediator for cognitive flexibility (28.7%) and inhibitory control (31.5%).

**Conclusion:**

The findings support the “Motor Coordination → Physical Fitness → Cognitive Function” pathway, highlighting PFI’s key mediating role. Integrated early interventions combining motor skill training and fitness enhancement are recommended to synergistically promote cognitive development.

## 1 Introduction

The preschool period (3–6 years) is a critical window for neurological development, motor skill formation, and cognitive function development in children (1, 2). This period is not only crucial for the consolidation of fundamental movement patterns but also represents a golden period for the rapid development of executive functions, including cognitive flexibility, inhibitory control, and working memory (3, 4). In recent years, with the rise of “embodied cognition” theory and developmental systems perspectives, researchers have increasingly focused on the bidirectional dynamic relationship between early physical experience and cognitive development in children (5, 6). Among these, motor coordination and physical fitness, as two core dimensions of physical ability, are believed to synergistically promote cognitive development through shared or complementary neurophysiological mechanisms (7, 8).

Motor coordination involves sensory integration, movement planning, and executive control, and its development relies on the plasticity of multi-brain region networks including the cerebellum, basal ganglia, and sensorimotor cortex (9, 10). Physical fitness, as a comprehensive reflection of cardiorespiratory endurance, muscular strength, and endurance, is closely related to cerebral blood flow, neurotransmitter balance, and the expression of brain-derived neurotrophic factors (11, 12). Individual differences in motor skills and physical fitness among preschool children not only affect their daily activity participation but may also influence the activation efficiency and functional connectivity of higher-order cognitive brain regions such as the prefrontal cortex through the “motor-cognitive coupling” mechanism (13, 14). Individual differences in motor skills and physical fitness among preschool children not only affect their daily activity participation but may also influence the activation efficiency and functional connectivity of higher-order cognitive brain regions such as the prefrontal cortex through the “motor-cognitive coupling” mechanism (15).

However, existing research has mostly examined the direct effects of motor coordination or physical fitness on cognition in isolation, overlooking their intrinsic connections during development and the potential “ability chain” they may constitute (16–18). Particularly noteworthy is that motor coordination is likely the foundation for children’s participation in moderate-to-vigorous physical activity, which in turn enhances physical fitness; improved fitness may then provide more adequate metabolic and neural resources to support cognitive activities, thereby more directly promoting cognitive function (19, 20). This hypothesized mediation pathway of “Motor Coordination (MC) → Physical Fitness (PFI) → Cognitive Function” has not been fully validated in preschool children.

Based on this, this study targeted children aged 3–6 years, aiming to systematically investigate the following questions: What are the associations of motor coordination and physical fitness with children’s cognitive flexibility, inhibitory control, and working memory? Does physical fitness mediate the relationship between motor coordination and these three cognitive functions? From the perspective of developmental windows, how can we understand the coordinated mechanism among motor coordination, physical fitness, and cognitive function, and its significance for early promotion?

## 2 Method

### 2.1 Participants

Sample size was calculated using G*Power v.3.1.9 with the following parameters: based on a moderate effect size (*f*^2^ = 0.15) commonly used in behavioral research (21), *α* = 0.05, power = 0.80, and up to 5 predictors in the regression model, the minimum required sample size was estimated to be 92. To account for potential attrition and clustering effects, we aimed to recruit a larger sample. Data collection took place from June to September 2025 in a kindergarten in Weifang City, Shandong Province. Following the principle of testing entire classes, 730 children were initially assessed. Seventeen were excluded due to incomplete key variable information, resulting in the final sample for analysis. Informed consent was obtained from all participants’ parents or legal guardians. The study was approved by the Institutional Review Board of the China Institute of Sport Science (Approval No.: CISSLA20250110).

### 2.2 Measures

#### 2.2.1 Motor coordination (MC)

The Movement Assessment Battery for Children, Second Edition (MABC-2), was used to effectively assess motor coordination development in preschool children. The MABC-2 has been widely validated for use in preschool populations and demonstrates good test–retest reliability (ICC = 0.85) and inter-rater reliability (ICC = 0.90) in this age group. The Chinese version with norms was available in China since 2016, providing an important reference for assessing children’s motor ability (22). The standardized assessment is divided into three age bands: 3–6 years, 7–10 years, and 11–16 years. Each age band has eight tasks belonging to three dimensions: manual dexterity, aiming and catching (body coordination), and balance. These three abilities, as fundamental components of motor development during growth, comprehensively assess various aspects of a child’s movement development level. Each age group has 8 test items, and the assessment takes approximately 30–40 min per child. Test scores were entered into the official accompanying system, and raw item scores were converted to standard scores considering age and gender. Standard scores for the three dimensions (manual dexterity, aiming and catching, balance) were obtained. Finally, these were standardized into a composite standard score reflecting overall motor coordination ability.

#### 2.2.2 Physical fitness index (PFI)

Before testing, instructions and demonstrations were given to the children. Six tests were administered to the preschool children: standing long jump, tennis ball throw, 10-meter shuttle run, 15-meter obstacle run, sit-and-reach, and walking on a balance beam (23). Scores from the six fitness indicators were standardized (Z-scores) within gender and age groups. These tests are part of the Chinese National Physical Fitness Measurement Standards Manual for Preschool Children (CPFS—preschool), which has established test–retest reliability (*r* = 0.78–0.92) and content validity for assessing physical fitness in this age group. Testing was conducted according to the detailed rules of the manual. Scores from the six fitness indicators were standardized (Z-scores) within gender and age groups.

#### 2.2.3 Cognitive function assessment

Cognitive function was assessed using behavioral tasks (24), with three validated paradigms measuring core executive function components: cognitive flexibility, inhibitory control, and working memory. All tasks were administered one-on-one by uniformly trained testers in a quiet room following standardized protocols.

(1) Cognitive flexibility

Measured using an iPad card sorting task adapted from the “Early Years Toolbox (25).” This task has been validated in preschool children, with good internal consistency (*α* = 0.82) and test–retest reliability (*r* = 0.79). In this task, children needed to sort stimuli according to changing rules. Specifically, a cartoon picture of a rabbit, either red or blue, appeared in the center of the screen, with red and blue “houses” displayed side-by-side below as target areas. Initially, children sorted according to a color rule (e.g., “put the red rabbit in the red house”). After a certain number of trials, the rule switched without warning to the opposite rule (e.g., “put the red rabbit in the blue house”). The task consisted of 24 trials: 8 pre-switch and 16 post-switch trials. The number of correct trials in the post-switch phase was recorded as the final score, with higher scores indicating better cognitive flexibility. This paradigm has shown good reliability and validity in preschool children.

(2) Inhibitory control

Inhibitory Control: Measured using the classic “Day/Night” Stroop-like paradigm (26). This task has been validated in preschool children, demonstrating adequate reliability (split-half reliability = 0.75) and convergent validity with other inhibitory control measures. Children were required to ignore the semantic meaning of pictures and name the opposite content according to instructions. Task materials included two types of cards: (1) “Day/Night” task: Children were presented with cards depicting a sun (representing day) or a moon and stars (representing night). They were instructed to say “night” when seeing the “day” card and “day” when seeing the “night” card. (2) “Happy/Sad” task: Children were presented with cards depicting a smiling face or a crying face. They were instructed to say “sad” when seeing the “happy” card and “happy” when seeing the “sad” card. Each task consisted of 16 trials (8 stimulus cards presented twice each in random order). The tester recorded the total number of correct inhibitory responses across all 32 trials. Higher scores indicated stronger inhibitory control.

(3) Working memory

Working Memory: Assessed using a self-designed “Self-Ordered Pointing Task (27), “primarily evaluating visual–spatial working memory span. This task was adapted from validated measures of working memory in preschoolers and showed acceptable internal consistency in our sample (*α* = 0.78). Test materials consisted of pictures of familiar objects (e.g., apple, car, ball). Procedure: The tester presented a test booklet page-by-page to the child. Each page displayed 3 to 8 non-repeating pictures in a random spatial layout. The tester first specified a target picture, and the child pointed to it. On the next page (with the same picture set but a changed layout), the tester specified a new target picture, and the child had to point to it while avoiding previously selected pictures. The task included 6 difficulty levels (number of pictures increasing from 3 to 8), with 2 trials per level, totaling 12 trials. A “two consecutive errors rule” was applied: testing stopped when the child made errors on both trials at a given difficulty level. The working memory score was the total number of correct new selections across all trials. Higher scores indicated better working memory ability. By changing the spatial arrangement of pictures on each page, the task effectively prevented reliance on positional memory, ensuring the valid assessment of the working memory component.

### 2.3 Testing procedure

All assessments were conducted during regular kindergarten hours in a quiet, dedicated testing room within the kindergarten. Each child was assessed individually by a trained tester. The assessment battery was administered in a fixed order: first the cognitive tasks (approximately 20–25 min), followed by the motor coordination assessment (MABC-2, approximately 30–40 min), and finally the physical fitness tests (approximately 20–25 min). A short break (5–10 min) was provided between each assessment block to minimize fatigue. The total testing time per child was approximately 1.5–2 h, spread across two sessions on separate days to avoid excessive burden. All testers underwent a standardized training program prior to data collection and followed a detailed testing manual to ensure consistency across participants.

### 2.4 Statistical analysis

All data analyses were performed using SPSS 27.0 and the PROCESS macro (version 3.5). First, descriptive statistics (mean, standard deviation) were calculated for continuous variables, and sample characteristics were reported separately by gender. Second, Pearson correlation analysis was used to examine bivariate relationships among motor coordination (MC), physical fitness (PFI), and the three cognitive functions (cognitive flexibility, inhibitory control, working memory). To explore the independent contributions of MC and PFI to cognitive function, hierarchical regression analyses were conducted, controlling for age, gender, and BMI. The first step (control layer) included age, gender, and BMI. The second step (predictor layer) simultaneously entered MC and PFI to assess their predictive power for cognitive function. Effect sizes (*β*) and their 95% confidence intervals were reported for all regression coefficients. Finally, to test the mediating role of PFI between MC and cognitive function, mediation analysis was performed using Hayes’ Bootstrap method (Model 4) via the PROCESS macro. This method does not rely on the normality assumption of the sampling distribution and has higher statistical power. The analysis was set with 5,000 resamples, and 95% bias-corrected confidence intervals (95% Bias-Corrected CI) were reported. The criterion for a significant mediation effect was a confidence interval for the indirect effect (ab) that did not include zero. The proportion of the effect was calculated as the ratio of the indirect effect to the total effect (c). This analysis was conducted controlling for age, gender, and BMI, constructing separate mediation models for each of the three cognitive functions as the dependent variable. To address the risk of Type I error due to multiple comparisons, we applied a Bonferroni correction for the three cognitive outcomes, setting the significance threshold at *p* < 0.017.

## 3 Results

### 3.1 Sample characteristics

The study included 713 preschool children, comprising 386 boys and 327 girls, all of whom completed all assessments. The sample age range was 3–6 years. Table 1 presents the descriptive statistics for the sample, stratified by gender.

**Table 1:** Sample characteristics by gender (*N* = 713).

Gender	Variable	*N*	Min	Max	Mean	SD
Male	Age	386	3	6	4.58	0.96
	Height (cm)	386	99.4	136.8	115.40	7.36
	Weight (kg)	386	13.7	40.6	20.87	4.08
	BMI	386	12.5	23.3	15.54	1.67
	MC	386	−13.22	8.12	−0.06	3.59
	PFI	386	−12.75	10.77	0.07	3.78
	Cognitive flexibility	386	0	12	4.73	4.06
	Inhibitory control	386	15	40	34.90	3.81
	Working memory	386	2	12	8.20	2.35
Female	Age	327	3	6	4.71	0.80
	Height (cm)	327	93.6	139.5	113.47	6.97
	Weight (kg)	327	12.2	40.6	19.85	4.00
	BMI	327	7.4	23.4	15.29	1.75
	MC	327	−9.09	7.52	0.08	3.30
	PFI	327	−12.27	8.81	−0.08	3.52
	Cognitive flexibility	327	0	12	5.33	4.13
	Inhibitory control	327	13	40	35.40	3.80
	Working memory	327	3	12	8.50	2.31

Values are presented as Mean (SD) unless otherwise specified. BMI, body mass index; MC, motor coordination composite standard score; PFI, physical fitness index z-score.

### 3.2 Correlation analysis

As shown in Table 2, Pearson correlation analysis indicated significant positive correlations among all main study variables (*p* < 0.01). Motor coordination (MC) showed a moderate positive correlation with physical fitness (PFI) (*r* = 0.443). MC was significantly positively correlated with all three cognitive functions, most strongly with cognitive flexibility (*r* = 0.250), followed by inhibitory control (*r* = 0.228) and working memory (*r* = 0.145). PFI also showed significant positive correlations with the three cognitive functions. Furthermore, the three cognitive functions were positively intercorrelated, with coefficients ranging from 0.203 to.241.

**Table 2:** Bivariate Pearson correlations between motor coordination, physical fitness, and cognitive function measures (*N* = 713).

Variable	MC	PFI	Cognitive flexibility	Inhibitory control	Working memory
MC	1	0.443^**^	0.250^**^	0.228^**^	0.145^**^
PFI	0.443^**^	1	0.241^**^	0.231^**^	0.189^**^
Cognitive flexibility	0.250^**^	0.241^**^	1	0.241^**^	0.203^**^
Inhibitory control	0.228^**^	0.231^**^	0.241^**^	1	0.213^**^
Working memory	0.145^**^	0.189^**^	0.203^**^	0.213^**^	1

MC, Motor Coordination; PFI, Physical Fitness Index. ^**^p < 0.01.

### 3.3 Regression analysis for cognitive functions

To explore the independent contributions of MC and PFI to cognitive function, hierarchical regression analyses were conducted separately for each cognitive function, controlling for age, gender, and BMI. Results are presented in Tables 3–5 with 95% confidence intervals for all effects.

**Table 3:** Hierarchical regression predicting cognitive flexibility (*N* = 713).

*X*	*Y*	*B*	SE	*β*	*t*	*p*	Δ*R*^2^	*F*	*p*(*F*)
Cognitive flexibility	Age	0.711	0.193	0.155	3.679	0.001			
	Gender	0.486	0.294	0.059	1.649	0.100			
	BMI	−0.057	0.086	−0.024	−0.666	0.506	0.100	12.965	<0.001
	MC	0.168	0.048	0.141	3.477	0.001			
	PFI	0.119	0.048	0.106	2.447	0.015			

MC, Motor Coordination; PFI, Physical Fitness Index. B, unstandardized coefficient; SE, standard error; *β*, standardized coefficient; CI, confidence interval.

**Table 4:** Hierarchical regression predicting inhibitory control (*N* = 713).

*X*	*Y*	*B*	SE	*β*	*t*	*p*	Δ*R*^2^	*F*	*p*(*F*)
Cognitive flexibility	Age	0.693	0.181	0.163	3.840	<0.001			
	Gender	0.415	0.275	0.054	1.507	0.132			
	BMI	0.019	0.080	0.008	0.234	0.815	0.090	9.836	<0.001
	MC	0.132	0.045	0.120	2.931	0.003			
	PFI	0.102	0.045	0.098	2.246	0.025			

MC, Motor Coordination; PFI, Physical Fitness Index. *B*, unstandardized coefficient; SE, standard error; *β*, standardized coefficient; CI, confidence interval.

**Table 5:** Hierarchical regression predicting working memory (*N* = 713).

*X*	*Y*	*B*	SE	*β*	*t*	*p*	Δ*R*^2^	*F*	*p*(*F*)
Cognitive flexibility	Age	0.458	0.112	0.176	4.089	<0.001			
	Gender	0.227	0.171	0.049	1.331	0.183			
	BMI	−0.067	0.050	−0.049	−1.343	0.180	0.062	3.456	0.032
	MC	0.023	0.028	0.035	0.835	0.404			
	PFI	0.059	0.028	0.093	2.107	0.035			

MC, Motor Coordination; PFI, Physical Fitness Index. B, unstandardized coefficient; SE, standard error; *β*, standardized coefficient; CI, confidence interval.

#### 3.3.1 Regression analysis for cognitive flexibility

As shown in Table 3, among the control variables, age (*β* = 0.155, *p* = 0.001) was a significant positive predictor. More importantly, both MC (*β* = 0.141, *p* = 0.001) and PFI (*β* = 0.106, *p* = 0.015) had independent, significant positive predictive effects on cognitive flexibility. The effects of gender and BMI were not significant.

#### 3.3.2 Regression analysis for inhibitory control

As shown in Table 4, MC and PFI also independently predicted inhibitory control. Age (*β* = 0.163, *p* < 0.001), MC (*β* = 0.120, *p* = 0.003), and PFI (*β* = 0.098, *p* = 0.025) were all significant positive predictors. The effects of gender and BMI were not significant.

#### 3.3.3 Regression analysis for working memory

For working memory, as shown in Table 5, age (*β* = 0.176, *p* < 0.001) was a stable positive predictor. PFI (*β* = 0.093, *p* = 0.035) significantly positively predicted working memory, while the independent predictive effect of MC was not significant (*β* = 0.035, *p* = 0.404).

### 3.4 Mediation effect analysis

**Figure 1:**
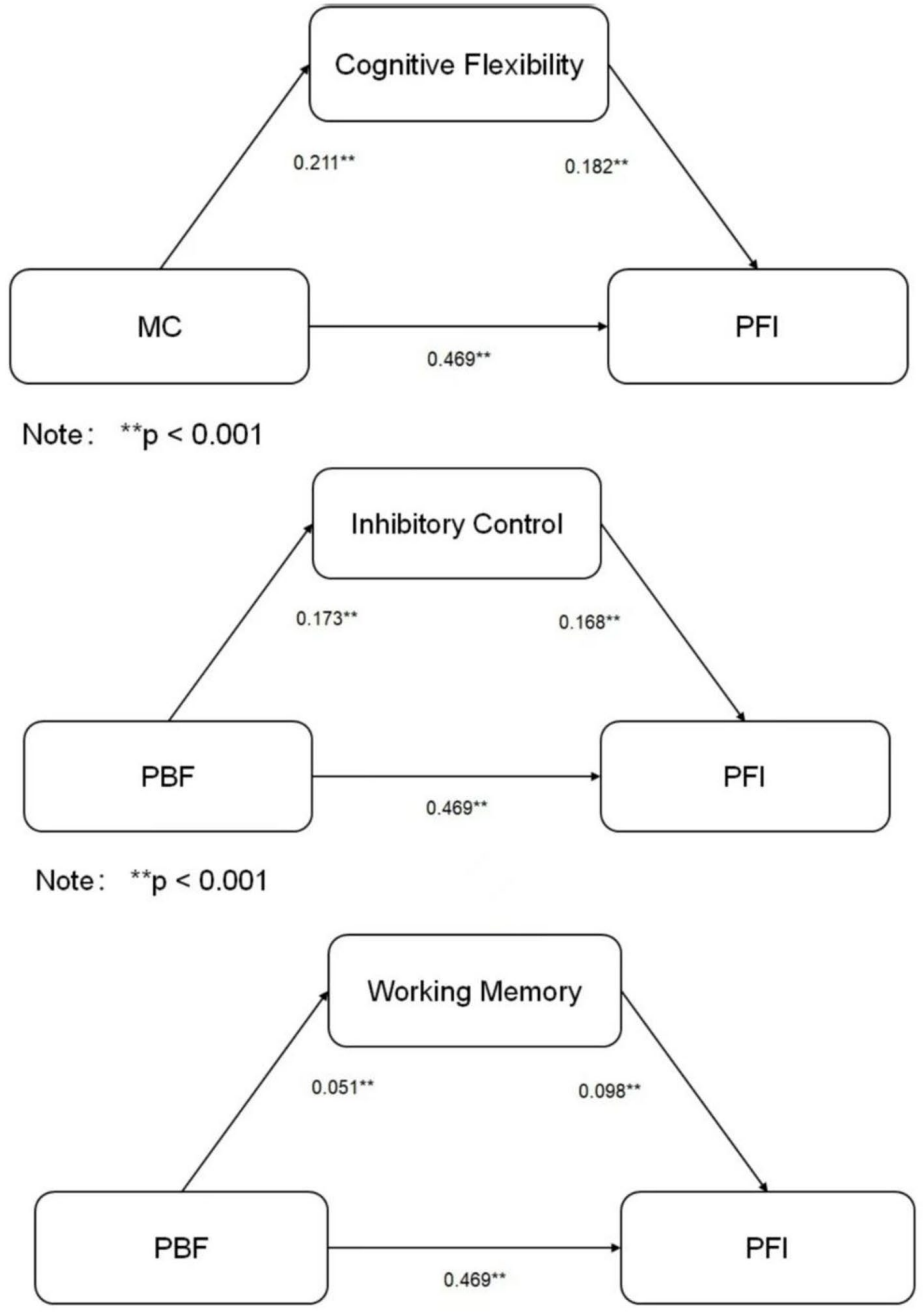
Mediation path diagrams for the three cognitive outcomes (*N* = 713). Values represent standardized coefficients (*β*). Solid lines indicate significant paths (*p* < 0.05); dashed line indicates non-significant path. MC, Motor Coordination; PFI, Physical Fitness Index. ^**^p < 0.01, *p* < 0.05.

To test the mediating role of PFI between MC and cognitive function, mediation analysis was conducted using the Bootstrap method (5,000 resamples). Results are shown in Table 6 and Figure 1.

**Table 6:** Mediating effects of PFI in the relationship between MC and cognitive functions (*N* = 713).

Variable	Path	Effect	95% CI	*β*	Effect proportion (%)
Cognitive flexibility	Total effect	0.296	[0.212, 0.380]	0.250	100%
	Direct effect	0.211	[0.118, 0.304]	0.178	71.3%
	Indirect effect	0.085	[0.045, 0.127]	0.072	28.7%
Inhibitory control	Total effect	0.251	[0.172, 0.330]	0.228	100%
	Direct effect	0.173	[0.085, 0.260]	0.157	68.5%
	Indirect effect	0.079	[0.041, 0.119]	0.072	31.5%
Working memory	Total effect	0.098	[0.048, 0.147]	0.145	100%
	Direct effect	0.051	[−0.003, 0.106]	0.076	53.1%
	Indirect effect	0.046	[0.020, 0.073]	0.069	46.9%

BC CI, bias-corrected confidence interval; *β*, standardized effect. All models controlled for age, gender, and BMI.

For cognitive flexibility, the total effect of MC was significant (*c* = 0.296, 95% CI [0.212, 0.380]). Mediation analysis showed that the indirect effect of MC on cognitive flexibility via PFI was significant (*ab* = 0.085, 95% CI [0.045, 0.127]), accounting for 28.7% of the total effect. The direct effect (*c*′ = 0.211) was also significant, indicating partial mediation by PFI.

For inhibitory control, the total effect of MC was significant (*c* = 0.251, 95% CI [0.172, 0.330]). The mediating effect of PFI was significant (*ab* = 0.079, 95% CI [0.041, 0.119]), accounting for 31.5% of the total effect, indicating partial mediation.

For working memory, the total effect of MC was significant (*c* = 0.098, 95% CI [0.048, 0.147]). The indirect effect through PFI was significant (*ab* = 0.046, 95% CI [0.020, 0.073]), accounting for 46.9% of the total effect. The direct effect of MC on working memory (*c*′ = 0.051, 95% CI [0.003, 0.106]) was not significant (as the CI includes 0, though the point estimate is positive; consistent with regression results), indicating that PFI served as a complete mediator.

## 4 Discussion

Through regression and mediation analyses, this study systematically revealed the intrinsic relationships among motor coordination, physical fitness, and cognitive function in preschool children and, for the first time in this age group, verified the key mediating role of physical fitness in the “motor coordination-cognitive function” relationship. These findings provide important evidence for understanding the mechanisms of early physical and mental coordination development and highlight the necessity of comprehensively promoting motor skills, fitness, and cognitive abilities during this critical developmental window.

First, the results indicate that both motor coordination and physical fitness have independent predictive effects on cognitive flexibility and inhibitory control (28). This suggests that during the preschool period, characterized by high neural plasticity, children’s movement experiences and physical adaptability may support prefrontal cortex-dominated cognitive control functions through different pathways (29, 30). We acknowledge that motor coordination and physical fitness share some conceptual overlap, as both involve aspects of balance and coordinated movement. However, the independent predictive effects observed in regression analyses suggest that each construct contributes uniquely to cognitive outcomes beyond shared variance. Motor coordination may more indirectly promote cognitive flexibility and inhibitory control by optimizing the efficiency of neural networks required for sensorimotor integration and movement planning (31); whereas physical fitness may more directly enhance the persistence and executive efficiency of cognitive tasks by improving cardiorespiratory function and cerebral oxygen supply (32). The two complement each other, forming a “twin-engine” supporting the development of children’s executive functions.

Second, for working memory, physical fitness showed an independent and stronger predictive power, while the influence of motor coordination was fully mediated by physical fitness. This pattern contrasts with that for cognitive flexibility and inhibitory control, suggesting that working memory—a function highly dependent on cognitive resources and neural-metabolic support—benefits more directly from good physical fitness (33, 34). Preschool children are in a period of rapid expansion of working memory capacity, and adequate bodily energy reserves and metabolic efficiency may be the physiological prerequisite for successfully completing information storage and processing tasks (27, 35). Therefore, during this early developmental window, “empowering” working memory development through enhanced physical fitness may be particularly significant.

More importantly, the “MC → PFI → Cognitive Function” mediation pathway revealed by this study provides empirical support for a mechanistic model of early physical-mental coordination development. This pathway indicates that motor coordination is not only the foundation for physical fitness development but also an indirect driving force for cognitive development; physical fitness acts as a “transformer” and “amplifier, “converting basic motor abilities into physiological resources usable by the cognitive system. However, we caution that these mechanistic interpretations are largely theoretical, as the proposed mechanisms (e.g., cerebral blood flow, neurotrophic factors) are derived from studies in older children and adults and were not directly assessed in the present study. This finding suggests that during the preschool stage, integrating motor skill training with aerobic physical activities could more effectively maximize cognitive promotion (36, 37).

From an educational and practical perspective, this study emphasizes the importance of fostering the coordinated development of movement, physical fitness, and cognition in preschool education and family activities. Isolated motor skill training or single-type fitness exercises may yield limited effects, whereas designing comprehensive physical activities that integrate coordinative challenges and aerobic load—such as obstacle runs, gamified circuit training, etc.—is more likely to effectively enhance children’s executive functions while promoting their physical fitness. Especially during the sensitive developmental period of 3–6 years, such integrated physical experiences may exert a profound.

## 5 Strengths and limitations

This study has several strengths. First, it focused on the preschool critical window, systematically examining the relationships among motor coordination, physical fitness, and cognitive function, providing empirical evidence for early integrated physical and mental development. Additionally, the relatively large sample size (*N* = 713) covering the entire 3–6 year age range enhances the representativeness and stability of the results.

However, limitations exist. First, the cross-sectional design precludes causal inference among variables. Although the mediation model is theoretically supported, longitudinal or experimental studies are needed for further validation. Second, cognitive function was assessed only through behavioral tasks. Second, the absence of an active control condition limits our ability to attribute observed associations specifically to motor coordination or physical fitness, as unmeasured confounders may influence results. Third, the sample was recruited from a single region in China, which may limit generalizability to other populations or settings. Fourth, cognitive function was assessed only through behavioral tasks, and the tasks used, while validated, may not capture the full complexity of executive functions. Fifth, the lack of long-term follow-up prevents examination of developmental trajectories. Sixth, while we conducted a sensitivity analysis excluding coordination-based fitness items, the potential for residual overlap between MC and PFI remains. Future research could incorporate neurophysiological measures like EEG or fNIRS to deepen understanding at the brain mechanism level. Subsequent studies could address these aspects to build a more comprehensive and dynamic model of early physical-mental development.

## 6 Conclusion

This empirical study found significant positive correlations between motor coordination, physical fitness, and cognitive function in preschool children. Both motor coordination and physical fitness independently predicted cognitive flexibility and inhibitory control. Physical fitness played a significant mediating role between motor coordination and all three cognitive functions. For working memory, physical fitness served as a complete mediator; for cognitive flexibility and inhibitory control, it served as a partial mediator. The results support the developmental pathway model of “Motor Coordination → Physical Fitness → Cognitive Function,” revealing the internal mechanism by which motor skills support cognitive development via enhanced physical fitness during the preschool period. However, these findings should be interpreted with caution given the cross-sectional design and the conceptual overlap between motor coordination and physical fitness measures.

In summary, the preschool stage is a critical window for the coordinated development of motor skills, physical fitness, and cognitive function. Future early intervention and educational practices should emphasize strategies integrating all three aspects. By designing physical activities that combine coordinative challenges, physical load, and cognitive demands, we can maximize the promotion of children’s holistic physical and mental development, laying a solid foundation for their subsequent learning and social adaptation.

## Data Availability

The raw data supporting the conclusions of this article will be made available by the authors, without undue reservation.
